# Development and Evaluation of Alternative Methods to Identify the Three Most Common Serotypes of *Salmonella enterica* Causing Clinical Infections in Kazakhstan

**DOI:** 10.3390/microorganisms9112319

**Published:** 2021-11-09

**Authors:** Sabyrkhan M. Barmak, Yuriy A. Sinyavskiy, Aidar B. Berdygaliev, Turegeldy Sh. Sharmanov, Irina S. Savitskaya, Gulmira T. Sultankulova, Elena V. Zholdybayeva

**Affiliations:** 1 Faculty of Biology and Biotechnology, Al-Farabi Kazakh National University, Almaty 050040, Kazakhstan; irasava_2006@mail.ru; 2 Biotechnology and Biologically Active Substances Laboratory, Kazakh Academy of Nutrition, Almaty 050008, Kazakhstan; Sinyavskiy@list.ru (Y.A.S.); berdaidar@mail.ru (A.B.B.); sabyr2103@gmail.com (T.S.S.); 3 Department of Pediatric Surgery, Asfendiyarov Kazakh National Medical University, Almaty 050012, Kazakhstan; arai_00.0@mail.ru; 4 National Center for Biotechnology, National Scientific Shared Laboratory of Biotechnology, Nur-Sultan 010000, Kazakhstan; lenazhol@gmail.com

**Keywords:** *Salmonella enterica*, *S.* Typhimurium, *S.* Enteritidis, *S.* Virchow, PCR, real-time PCR, RAPD-PCR

## Abstract

In this study, we aimed to compare the performance of conventional PCR and real-time PCR assays as screening methods for identification of three frequent, clinically significant Salmonella serovars in Kazakhstan. We determined the diagnostic efficacy of three molecular methods for detection of *S. enterica* subsp. enterica and typing *S.* Typhimurium, *S.* Enteritidis, and *S.* Virchow. A total of 137 clinical samples and 883 food samples were obtained in Almaty in 2018–2019. All tests showed high analytical specificity for detecting *S. enterica* and its corresponding serovariants (100%). The sensitivity of real-time PCR for each of the tested targets was 1–10 microbial cells and in conventional PCR 10–100 microbial cells. The trials with conventional PCR and real-time PCR had a diagnostic efficacy (DE) of 100% and 99.71%, respectively. The DE of real-time PCR and conventional PCR for detecting *S.* Enteritidis and *S.* Typhimurium was 99.90%, while the DE of conventional PCR and real-time PCR for detecting *S.* Virchow was 99.31% and 99.80%, respectively. The RAPD-PCR analysis of the genomic DNA of *Salmonella enterica* showed the genetic kinship of *S.* Enteritidis isolates, and the genetic heterogeneity of *S.* Typhimurium and *S.* Virchow isolates. Thus, the developed methods can be considered as alternatives to classical serotyping using antisera.

## 1. Introduction

The sickness rate of salmonellosis remains one of the most pressing health problems in many countries of the world. Salmonellosis occurs in both developed and developing countries [1,2,3]. In the etiology of bacterial intestinal infections in humans, *Salmonella enterica* subsp. *enterica* takes the leading place. To date, over 2500 *Salmonella enterica* serovars have been registered [4,5]. Many serotypes of *S.* *enterica* are pathogenic to both animals and humans. Serovars *S*. Typhimurium and *S*. Enteritidis are the most common causes of human salmonellosis in many countries of the world [6,7], including Kazakhstan [8]. However, in some regions, other serovars are more important [9].

Outbreaks of salmonellosis are associated with the consumption of contaminated food, which often leads to serious illnesses that may require hospitalization or even death. *Salmonella* contamination of food can occur at any stage of production and distribution and therefore requires continuous and effective monitoring of *Salmonella* in the production chain using rapid, effective, and proven methods. In addition, timely identification and typing of *Salmonella* is critical for the epidemiological surveillance of Salmonellosis and tracing the source of the outbreak [10]. 

Until now, the gold standard for the diagnosis of salmonellosis is the microbiological method (isolation of *Salmonella* spp. from feces, blood, vomit, urine, bile, blood). However, the long waiting period for results due to the need to quickly conduct appropriate antibiotic therapy is undoubtedly a disadvantage of this method. The situation is compounded by the fact that many patients are treated with antibiotics before symptoms of sepsis develop. In such cases, blood cultures are very difficult to perform since they contain antibiotics that inhibit the growth of microorganisms. Therefore, the detection of microbial nucleic acids is promising for efficient, accurate, and rapid diagnosis of salmonellosis. In addition, the sensitivity of molecular methods is higher than the sensitivity of the culture method, and the preliminary use of antibiotics does not affect the test results. Thus, there is a need to develop, validate, and implement alternative methods for the detection of *Salmonella*. 

This study aimed to develop alternative molecular methods (PCR, RT-PCR, RAPD PCR) for the detection and typing of *Salmonella* in clinical samples and food products and their assessment on clinical samples from sick patients and food products collected in Almaty in 2018–2019.

## 2. Materials and Methods

### 2.1. Microorganisms Strains

Reference strains of *Salmonella* and other bacteria were used to determine the specificity of the reaction: *S*. Enteritidis (*S.*e-0071), *S*. Typhimurium TA 98 (reference strain), *S*. Typhimurium (*S.*t-0072), *S.* Virchow (reference strain), *S*. Infantis (*S.*i-0073), *S.* Abortusovis *37*, *S. Gallinarum 65*, *S.* Abortus equi *17*, *S.* Cholera-suis *51*, *S.* Dublin *31*, *Pasteurella multocida* subsp. *multocida* (ATCC 10544), *Clostridium perfringens* Strain S 107 (ATCC 13124), *Clostridium sporogenes NCTC 532* (ATCC-19404), *Escherichia coli* (ATCC 25922), *Bacillus cereus* (ATCC 11778), *Bacillus subtilis* (ATCC-6633), *Staphylococcus aureus* (ATCC 25923), *Staphylococcus aureus* (ATCC-6538P), *Pseudomonas aeruginosa* Strain Boston *41501* (ATCC 27853), *Pseudomonas aeruginosa* (ATCC-9027), *Candida albicans* 3147 (ATCC-10231), *Mycoplasma hyorhinis BTS-7* (ATCC-17981), *Mycoplasma gallisepticum* (ATCC-19610), *Mycoplasma synoviae WVU 1853* [NCTC 10124] (ATCC-25204), *Klebsiella pneumoniae* (ATCC 13883), *Aspergillus brasiliensis* (ATCC-9642) formerly identified as *A. niger*.

### 2.2. Salmonella Isolates Used for PCR Method Validation

The validation of the PCR method included the analysis of 1020 samples (883 samples from food products and 137 clinical samples) collected in Almaty (Table 1). Samples of various food products were randomly collected from retail markets in Almaty between May 2018 and April 2019. In large shopping centers, sufficient attention is paid to food safety; therefore, the bulk of the food products were purchased from various markets in Almaty for research purposes. Samples were collected and prepared according to the recommendations [11,12].

The collection of clinical material (stool) of a patient suffering from an acute intestinal infection was carried out by doctors at the Children’s City Clinical Infectious Disease Hospital in Almaty in 2018–2019. Clinical samples were selected and transported following the requirements noted in the sanitary rules [13]. A total of 137 clinical samples were collected from patients for research.

Each sample was labeled, placed in a sterile plastic sample bag, transported to the laboratory on ice, and processed immediately.

### 2.3. Isolation and Identification of Salmonella

Isolation and identification of *Salmonella* were performed using standard methods described in regulatory documents [14,15,16].

### 2.4. DNA Extraction

Bacterial genomic DNA was extracted using the DNeasy Blood & Tissue Kit (Qiagen, Hilden, Germany). The concentration and purity of the extracted genomic DNA were measured on a MaestroNano^®^ spectrophotometer (Maestrogen, Las Vegas, NV, USA).

### 2.5. Specific Primers and Probes

Specific PCR primers and a probe for typing *S.* Enteritidis have been developed for the gene encoding the protein Prot6e of fimbrial biosynthesis located at a specific site of the 60-kb plasmid of virulence of *S.* Enteritidis (U66901.1) [17].

The mdh gene encoding the *S.* Typhimurium malic acid dehydrogenase enzyme is conservative and was chosen for the development of specific primers and a real-time PCR probe (X61029.1) [18].

Specific PCR primers and a probe for typing *S.* Virchow were designed for the CRISPR gene located in the conservative region of the 100–1400 bp CRISPR gene (KF931137.1) [19].

The primers were designed using the Vector NTI Suite 9 software (Invitrogen, Carlsbad, CA, USA) and tested using BLAST to confirm their specificity (Table 2).

### 2.6. Real-Time Polymerase Chain Reaction

Real-time PCR was performed by TaqMan technology using a Rotor-Gene Q thermal cycler (Qiagen, Hilden, Germany). When performing real-time PCR, Platinum SuperFi PCR Master Mix (Invitrogen, Waltham, MA, USA) was amplified with primers and probes specific for *Salmonella enterica* bacteria and its types: *S.* Enteritidis*, S.* Typhimurium*,* and *S.* Virchow (Table 1).

The RT-PCR mixture consisted of 2.5 µL of 10× AccuPrime PCR Buffer II; 1 μL of each (10 pmol) primers; 1 μL of (5 pmol) Probe-FAM; 0.5 μL of AccuPrime *Taq* DNA Polymerase; 1 μL of DNA; up to 25 μL of water. The thermocycling protocol was 95 °C for 3 min; 94 °C for 20 s, 57 °C (for *S. enterica*); 60 °C (for *S.* Enteritidis); 62 °C (for *S.* Typhimurium); 59 °C (for *S.* Virchow) for 30 s for a total 45 cycles.

### 2.7. PCR

The conventional PCR was performed with Platinum SuperFi PCR Master Mix (Invitrogen, Waltham, MA, USA) and specific primers for *S. enterica* and its types: *S.* Enteritidis, *S.* Typhimurium, and *S.* Virchow (Table 1). The production of specific DNA regions of *Salmonella* bacterium was carried out in a Mastercycler Pro thermal cycler (Eppendorf, Hamburg, Germany). The following is the reaction composition for amplification of bacterial DNA fragment: 2.5 µL of 10× buffer; 1 μL of dNTPs; 2 μL of MgCl_2_; 1 μL of each (20 pmol) primers; 0.5 μL of *Taq* DNA Polymerase; 3 μL of DNA; up to 25 μL of water. The thermal cycling conditions were 94 °C for 5 min; 95 °C for 30 s, 55 °C (for *S. enterica*); 59 °C (for *S.* Enteritidis); 59 °C (for *S.* Typhimurium); 60 °C (for *S.* Virchow) for 30 s, 72 °C for 1 min for a total of 35 cycles; 72 °C for 7 min; stored at 4 °C.

### 2.8. Randomly Amplified Polymorphic DNA (RAPD) PCR

RAPD PCR mixtures contained the following components: 2.5 µL of 10× buffer; 1 μL of dNTPs; 1 μL of MgCl_2_; 2 μL of primer; 0.5 μL of *Taq* DNA polymerase; 5 μL of DNA; up to 25 μL of water. The primer RAPD-A 5′GCG GGA ATG CTG AAG ATA AG3′ was used to amplify DNA (Table 1). PCR conditions were as follows: 94 °C for 5 min; 94 °C for 45 s, 35 °C for 5 s, 72 °C for 1.20 min for a total of 40 cycles; 72 °C for 10 min.

### 2.9. Electrophoretic Analysis of DNA Amplification Products

All RAPD PCR products were separated by horizontal electrophoresis (BioRad, Munich, Germany) on a 1.5% agarose solution in Tris-acetate-EDTA buffer. The results are documented by photographing gels in the BioRad gel documenting the system.

### 2.10. Statistical Analysis

When determining the performance indicators of laboratory tests, true positive (TP), true negative (TN), false positive (FP), and false negative (FN) research results were used.

The calculations are based on the following formulas: sensitivity (SN) = (TP/TP + FN), specificity (SP) = (TN/TN + FP), positive predictive value PPV = (TP/TP + FP), negative predictive value (NPV) = (TN/TN + FN), diagnostic efficacy (DE) = (TP + TN/TP + FP + FN + TN) [20]. Ninety-five percent confidence interval (95% CI) was evaluated by Wilson’s calculation method [21].

## 3. Results

### 3.1. Specificity, Sensitivity, and Efficacy of Real-Time PCR and Conventional PCR

**Figure 1:**
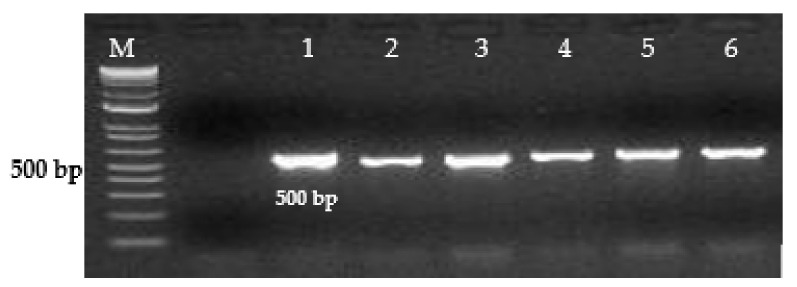
Detection of *S. enterica* using PCR primers SE Inv-1F and SE Inv-1R (500 bp). Lane M—1kb ladder (Invitrogen). Lane 1—PCR products amplified from *S.* Enteritidis (*S.* e-0071), lane 2—PCR products amplified from *S.* Typhimurium TA 98, reference strain, lane 3—PCR products amplified from *S.* Virchow, reference strain, lanes 4, 5, 6—clinical samples, positive for *S. enterica.*

**Figure 2:**
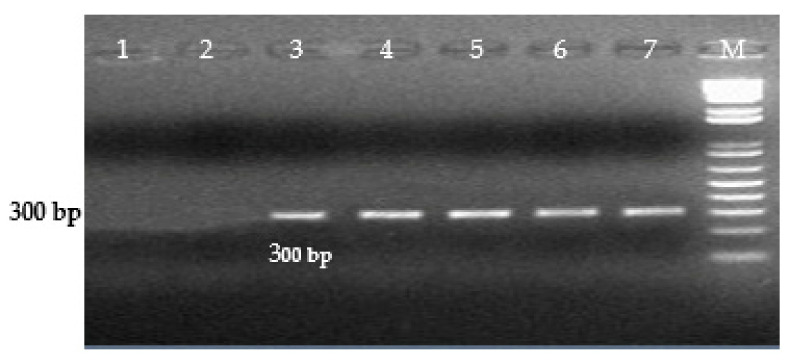
Detection of *S.* Enteritidis using PCR primers SE Prot6e-1F and SE Prot6e-1R (300 bp). Lane M—1kb ladder (Invitrogen). Lane 1—*S.* Typhimurium (S.t-0072), lane 2—*S.* Virchow (reference strain), lane 3—PCR products amplified from *S.* Enteritidis (S.e-0071) (positive control), lanes 4, 5, 6, 7—clinical samples, positive for *S.* Enteritidis.

**Figure 3:**
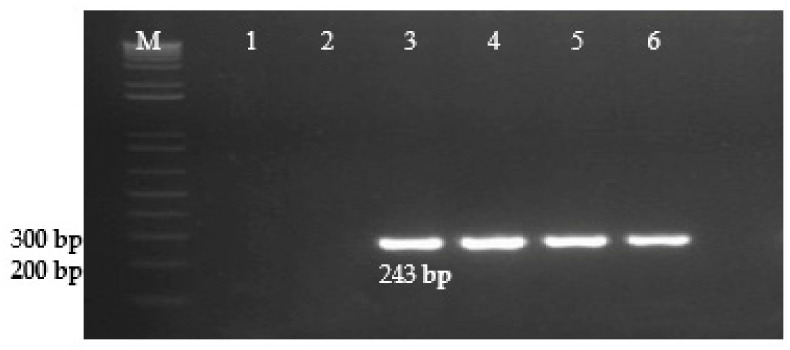
Detection of *S.* Typhimurium using PCR primers ST mdh-1F and ST mdh-1R (243 bp). Lane M—1kb ladder (Invitrogen). Lane 1—PCR products amplified from *S.* Enteritidis (S.e-0071), lane 2—*S.* Virchow (reference strain), lane 3—PCR products amplified from *S.* Typhimurium (S.t-0072) (positive control), lanes 4, 5, 6—clinical samples, positive for *S.* Typhimurium.

**Figure 4:**
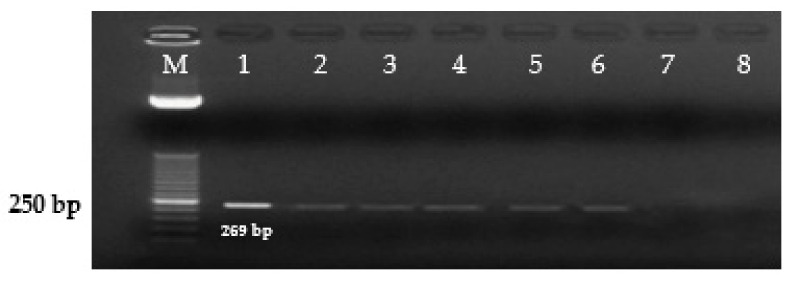
Detection of *S.* Virchow using PCR primers SV CRISPR–1F and SV CRISPR–1R (269 bp). Lane M—50 bp ladder (Invitrogen). Lane 1—PCR products amplified from *S.* Virchow, reference strain (positive control). Lanes 2, 3, 4, 5, 6—clinical samples, positive for *S.* Virchow*,* lane 7—*S.* Enteritidis (S.e-0071), lane 8—*S.* Typhimurium (S.t-0072).

The diagnostic primers developed in this study for the Inv gene of *S. enterica* amplified a 500 bp DNA fragment. (Figure 1), for the Prot6e gene, *S.* Enteritidis amplified a DNA fragment of 300 bp. (Figure 2), for the mdh gene, *S.* Typhimurium amplified a DNA fragment of 243 bp. (Figure 3), for the CRISPR gene, *S.* Virchow amplified a 269 bp DNA fragment (Figure 4).

The specificity of conventional PCR and real-time PCR was confirmed by testing the *S. enterica* bacterium, its serotypes, and non-salmonella microorganisms (Table 3). The specificity of the oligonucleotides and probes used was first confirmed by testing on a panel of 10 *Salmonella* control organisms, and then expanded to testing a panel of 34 *Salmonella* isolates isolated from food samples and 65 *Salmonella* isolates isolated from clinical samples. Both tests showed high analytical specificity in detecting *S*. *enterica* and its serotypes. No cross-reaction was observed when determining the affiliation of strains and isolates to *S*. Enteritidis, *S*. Typhimurium, and *S*. Virchow in both conventional and real-time PCR.

The limit of detection (LOD) was calculated by amplifying a series of 10-fold dilutions of *Salmonella* bacterium DNA extracts. The LOD for detection at the threshold of real-time PCR results was 100 copies/mL of target sequence. The linear measurement range was 100–10,000,000 copies/mL of the target sequence. The analytical sensitivity of classical PCR was 10–100 microbial cells.

To estimate the diagnostic efficacy of real-time PCR and conventional PCR methods in detecting *S. enterica* and its serotypes, 1020 biological samples (883 samples from food products and 137 samples of clinical sample) were analyzed (Table 4 and Table 5).

Of the 1020 samples obtained from clinical samples, food raw materials, and food products, *S. enterica* were detected in 99 (9.70%) by real-time PCR. The same results were obtained using cultivation methods. Conventional PCR detected *S. enterica* in 96 (9.41 %) samples. The diagnostic efficacy of real-time PCR in the detection of *S. enterica* was 100, while for the conventional PCR was 99.71%.

Real-time PCR detected *S.* Enteritidis in 20 (1.96%) samples, *S.* Typhimurium in 42 (4.12%), and *S*. Virchow in 24 (2.35%) out of 1020 samples in parallel with the conventional PCR, in which *S.* Enteritidis was detected in 20 (1.96%) samples*, S.* Typhimurium in 42 (4.12%), and *S.* Virchow in 19 (1.86%). The diagnostic efficacy of real-time PCR and conventional PCR for the detection of *S.* Enteritidis and *S.* Typhimurium was 99.90%. The diagnostic efficacy of real-time PCR for detection of *S.* Virchow was 99.80%, and conventional PCR was 99.31%.

### 3.2. Typing of Salmonella enterica Strains by RAPD PCR

To identify the genetic diversity of *S.* Enteritidis strains (13 isolates from clinical sample and 8 isolates from food), *S.* Typhimurium (29 isolates from the clinical samples and 14 isolates from food), and *S.* Virchow (23 isolates from clinical sample and 3 isolates from food), a PCR analysis was performed using a RAPD primer. The PCR analysis of the genomic DNA of *Salmonella enterica* bacteria using RPC primers showed the heterogeneity of the studied strains (Table 6).

The same specific set of DNA fragments were obtained with the RAPD primer for all the studied *S.* Enteritidis isolates. For all isolates extracted from both clinical samples and food products (group A), 6 amplicons were identified with a length of approximately 250, 350, 650, 1000, 1250, and 3000 bp, which indicates their genetic relationship.

When genotyping *S.* Typhimurium strains, 4 amplicons (200, 350, 1000, 1250 bp) were detected in 12 isolates extracted from food products and in all 29 studied isolates extracted from clinical sample (group B), and 3 amplicons (200, 350, 1000 bp) were obtained for 2 isolates extracted from food products (group C).

The results of *S*. Virchow strain DNA amplification with RAPD-primer showed that for all isolates extracted from food products, 3 amplicons were identified, with lengths of approximately 200, 650, and 1200 bp (group D), which indicates their genetic relationship. 

In the study of *S.* Virchow isolates extracted from clinical samples, three groups with different numbers of amplicons were identified. In group I, 19 isolates were identified (3 amplicons with a length of approximately 300, 650, 1200 bp), in group F, 2 isolates (2 amplicons with a size of 400, 650 bp), and in group G2 isolates (4 amplicons with a size of 300, 500, 650, 1200 bp). The results show that *S.* Virchow isolates obtained from the clinical sample are genetically diverse.

## 4. Discussion

Rapid and effective diagnosis of food pathogens continues to be a serious public health problem. Monitoring the presence of foodborne pathogens is a key condition for identifying potential problems in the production, processing, and preparation of food products or the process of sales. The classical methods of detecting *Salmonella* used to date are time-consuming and take 3 to 5 days to complete. Alternative methods based on the detection of nucleic acids are still not applicable for wide usage. One of the reasons is that the developed methods have not passed validation tests.

All clinical patients in Kazakhstan are tested in regard to the current surveillance program for the presence of *Salmonella* with the use of standard methods based on the isolation of cultures with the following identification by biochemical and serological methods. Molecular identification methods are rarely used. It should also be noted that there are few reports of *Salmonella* contamination of food products in Kazakhstan. This indicates the lack of studies of food products for *Salmonella* contamination. Therefore, *Salmonella* contamination of food products in the retail trade should be solved by constant monitoring and control using modern methods.

This study shows the results of the improvement of conventional PCR and real-time PCR for detecting *S. enterica* and its serotypes. There are quite a few methods applied to indicate and identify *Salmonella* bacteria [22,23]. Various genes are known to act as genetic markers of *Salmonella*. Several reports have been published on the use of the PCR method to detect *S. enterica* targeting the invA gene [22,23,24,25]. In our studies, primers targeting the invA gene were designed for both tests to detect *S. enterica*. Previously, the PCR targeting of the InvA gene for *S. enterica* showed 100% specificity when testing 94 *Salmonella* strains (inclusiveness) and 32 non-*Salmonella* strains (exclusivity) [26]. The conventional PCR and real-time PCR conducted with our primers on the invA gene allowed us to obtain similar results. Both tests showed a fairly high specificity for *S. enterica*. The sensitivity of real-time PCR for each of the tested targets was 1–10 microbial cells, and in classical PCR, 10-100 microbial cells. The sensitivity of real-time PCR for each of the tested targets was 1-10 microbial cells, while in conventional PCR, it was 10–100 microbial cells. 

The choice of specific target genes is crucial for *Salmonella* serotyping. Despite the high homology among serovariants, it was found that some genes are associated with specific serovars. Thus, it was established that the genes Prot6e [17], mdh [18], CRISPR [19], spvA [27], rfb [28], Sdf-1 [23] fliC [28,29], SefA [30], invA [31], fimA [32], and ipaJ [33], are suitable for the specific detection and serotyping of *Salmonella* in various clinical samples. As noted in Section 2.5, the site of the Prot6e gene was selected for the detection of *S.* Enteritidis, the site of the mdh gene for the detection of *S.* Typhimurium, and the site of the CRISPR gene for the detection of *S.* Virchow. In silico analysis revealed no mismatch between primers and probes with the available bacterial genome in GenBank. Both tests showed a fairly high specificity of *S. enterica* serotypes. All *S.* Enteritidis isolates tested in this study were positive with Prot6e reagents, *S.* Typhimurium with mdh reagents, and *S.* Virchow with CRISPR reagents, as expected.

The performance of the developed PCR methods was verified on clinical samples and food samples collected in 2018–2019 in Almaty. The results of real-time PCR in the study of clinical samples and food samples demonstrated an excellent correlation with the cultivation method. Therefore, examination of clinical samples showed that in 65 (47.7%) out of 137 samples were positive results in real PCR; at the same time, out of 883 studied food samples, only 34 (3.85%) were PCR-positive. Similar results were obtained using the classical method (cultivation). The high proportion of PCR-positive results among the studied clinical samples may be due to the fact that clinical samples were taken only from patients hospitalized with acute intestinal infections. In the study of clinical and food samples, in all cases except real-time PCR on *S. enterica*, from 1 to 3 false negative results were obtained. Perhaps this is due to the fact that some *Salmonella* strains have natural mutations in the loci used in PCR as targets, which can lead to false negative results [34,35]. To prove this hypothesis, it is necessary to sequence target loci in these strains. The diagnostic efficacy of real-time PCR and PCR for the detection of *S. enterica* was 100% and 99.71%, respectively. The diagnostic efficacy of real-time PCR and PCR for the detection of *S*. Enteritidis and *S*. Typhimurium was the same 99.90%, while the diagnostic efficacy of PCR and real-time PCR for the detection of *S*. Virchow were 99.31% and 99.80%, respectively.

The information about the origin of new species of *S. enterica* in various countries can help in the identification and tracking of new emerging pathogens. It is possible now to determine the serotypes of *Salmonella* that prevail in Kazakhstan with the help of the developed PCR methods. As a result of the study of 1020 biological samples (883 samples from food products and 137 clinical samples) collected in 2018-19 in Almaty, 99 isolates were identified by the developed PCR methods and isolated by the cultivation method. Of these, 21 (21.2%) isolates are classified as *S*. Enteritidis, 43 (43.4%) isolates as *S*. Typhimurium, and 26 (26.3%) isolates as *S*. Virchow. Earlier, it was shown that two serotypes prevail in Kazakhstan: *S.* Typhimurium and *S.* Enteritidis [8]. One of the focuses of this study was to show that *S.* Virchow is also a serotype that is common to Kazakhstan. To confirm this, it is necessary to conduct additional studies on a larger number of clinical samples and food products.

The performed molecular analysis using RAPD PCR to identify the genetic diversity of *S. enterica* bacterial isolates obtained from the food chain and clinical samples showed the genetic relationship of *S.* Enteritidis isolates and the genetic heterogeneity of *S.* Typhimurium and *S.* Virchow isolates.

## 5. Conclusions

The research results demonstrate that the PCR testing platforms and methods are sensitive and specific, which makes these methods valuable tools for detecting *S.* Enteritidis, *S.* Typhimurium, and *S.* Virchow directly in food samples and clinical material. The developed PCR methods meet the requirements of diagnostic PCR, and after further interlaboratory validation studies, can become standardized methods for rapid detection of *Salmonella* in diagnostic laboratories. 

## Figures and Tables

**Table 1:** List of biological samples collected in 2018–2019 in Almaty for the isolation of *Salmonella.*

*n*	Name	Number of Tested Samples		Number of Isolated *Salmonella* Isolates	
		2018	2019	2018	2019
1	Clinical samples	137	0	65	0
2	Meat and meat products	63	55	7	1
3	Fish and fish products	38	32	1	4
4	Vegetables	67	35	1	0
5	Berries	23	17	0	0
6	Bird	35	34	8	2
7	Milk and dairy products	94	65	3	1
8	Mushrooms	27	16	0	0
9	Salads	20	13	0	0
10	Dried fruits	36	25	0	0
11	Fruits	24	27	0	0
12	Confectionery	23	21	0	0
13	Eggs	50	43	5	1
Total		637	383	90	9

**Table 2:** Oligonucleotide primers and probes for identification of *Salmonella enterica* species and their types in real-time PCR and PCR.

Type	Primer or Probe	Sequence	PCR Product Size
*Salmonella enterica*	SE-F	AGGTGACGCTATTGCCGGCAT	155
	SE-R	ATGCGGGGATCTGGGCGA	
	SE-Probe	FAM-ATTTCGGTGGGGATGACTCGCCAT-BHQ-1	
*S.* Enteritidis	SEE-F	CGTCGTTGCTGCTTCCGGGA	176
	SEE-R	GCTACAGAGAGTCACACTAA	
	SEE-Probe	FAM- TGCTGTAGATGCAAGGGTGCCTAA-BHQ-1	
*S.* Typhimurium	SET-F	GAAGTTGAAGTGCCGGTGAT	251
	SET-R	CATTCCACCACGCCCTTCT	
	SET-Probe	FAM- CAGATTCCAGGCGTAAGTTTTA-BHQ-1	
*S.* Virchow	SEV-F	ACACCAGTACGACGATCTGCG	105
	SEV-R	ATAAACCGGGCAACTGGG	
	SEV-Probe	FAM-GGAACACATAAACAGCGCCCAGAT-BHQ-1	
*Salmonella enterica*	SE Inv-1F	GTGAAATTATCGCCACGTTCGG	500
	SE Inv-1R	ATCGCCATTTACGCGGGTCA	
*S.* Enteritidis	SE Prot6e-1F	TAACCGGAGAGGCGCTCATC	300
	SE Prot6e-1R	AACCATGCTCAGCTGCTCCA	
*S.* Typhimurium	ST mdh-1F	GTGCCGGTGATTGGCGGGCA	243
	ST mdh-1R	CGCATTCCACCACGCCCTTC	
*S.* Virchow	SV CRISPR–1F	GATCTGCGCGAACAATATCA	269
	SV CRISPR–1R	CCGTTGTACTGATCATCTTC	
*S.* Enteritidis *S.* Typhimurium *S.* Virchow	RAPD-A	GCGGGAATGCTGAAGATAAG	–

Note: All oligonucleotides have been developed in the framework of this research.

**Table 3:** Diagnostic specificity of conventional PCR and real-time PCR.

Control Organism	Real-Time PCR				Conventional PCR			
	*S. enterica*	*S.* Enteritidis	*S.* Typhimurium	*S.* Virchow	*S. enterica*	*S.* Enteritidis	*S.* Typhimurium	*S.* Virchow
*S.* Enteritidis (S.e-0071)	Pos	Pos	Neg	Neg	Pos	Pos	Neg	Neg
*S.* Typhimurium TA 98 (reference strain)	Pos	Neg	Pos	Neg	Pos	Neg	Pos	Neg
*S.* Typhimurium (S.t-0072)	Pos	Neg	Pos	Neg	Pos	Neg	Pos	Neg
*S.* Virchow (reference strain)	Pos	Neg	Neg	Pos	Pos	Neg	Neg	Pos
*S.* Infantis (S.i-0073)	Pos	Neg	Neg	Neg	Pos	Neg	Neg	Neg
*S. A*bortusovis 37	Pos	Neg	Neg	Neg	Pos	Neg	Neg	Neg
*S.* Gallinarum 65	Pos	Neg	Neg	Neg	Pos	Neg	Neg	Neg
*S.* Abortus equi 17	Pos	Neg	Neg	Neg	Pos	Neg	Neg	Neg
*S.* Cholera suis 51	Pos	Neg	Neg	Neg	Pos	Neg	Neg	Neg
*S.* Dublin 31	Pos	Neg	Neg	Neg	Pos	Neg	Neg	Neg
*Pasterella multocida* subsp*. multocida* (ATCC-10544)	Neg	Neg	Neg	Neg	Neg	Neg	Neg	Neg
*Clostridium perfringens* Strain S 107 (ATCC-13124)	Neg	Neg	Neg	Neg	Neg	Neg	Neg	Neg
*Clostridium sporogenes* NCTC 532 (ATCC-19404)	Neg	Neg	Neg	Neg	Neg	Neg	Neg	Neg
*Escherichia coli* (ATCC-25922)	Neg	Neg	Neg	Neg	Neg	Neg	Neg	Neg
*Bacillus cereus* (ATCC-11778)	Neg	Neg	Neg	Neg	Neg	Neg	Neg	Neg
*Bacillus subtilis* subsp. *spizizenii* (ATCC-6633*)*	Neg	Neg	Neg	Neg	Neg	Neg	Neg	Neg
*Staphylococcus aureus* (ATCC-25923)	Neg	Neg	Neg	Neg	Neg	Neg	Neg	Neg
*Staphylococcus aureus* subsp. *aureus* (ATCC-6538P)	Neg	Neg	Neg	Neg	Neg	Neg	Neg	Neg
*Pseudomonas aeruginosa* Strain Boston 41501 (ATCC-27853)	Neg	Neg	Neg	Neg	Neg	Neg	Neg	Neg
*Pseudomonas aeruginosa* (ATCC-9027*)*	Neg	Neg	Neg	Neg	Neg	Neg	Neg	Neg
*Candida albicans;* 3147 *(*ATCC*-*10231)	Neg	Neg	Neg	Neg	Neg	Neg	Neg	Neg
*Mycoplasma hyorhinis;* BTS-7 (ATCC-17981)	Neg	Neg	Neg	Neg	Neg	Neg	Neg	Neg
*Mycoplasma gallisepticum* (ATCC-19610)	Neg	Neg	Neg	Neg	Neg	Neg	Neg	Neg
*Mycoplasma synoviae;* WVU 1853 [NCTC 10124] (ATCC-25204)	Neg	Neg	Neg	Neg	Neg	Neg	Neg	Neg
*Klebsiella pneumoniae* (ATCC-13883)	Neg	Neg	Neg	Neg	Neg	Neg	Neg	Neg
*Aspergillus brasiliensis; formerly A. niger* (ATCC-9642)	Neg	Neg	Neg	Neg	Neg	Neg	Neg	Neg

Pos, positive. Neg, negative. All laboratory isolates were sequenced using 16S RNA.

**Table 4:** Identification of *Salmonella enterica* and its types *S.* Enteritidis*, S.* Typhimurium, and *S.* Virchow in samples using various tests.

	Result	True Positive (TP)	False Positive (FP)	False Negative (FN)	True Negative (TN)	Total
Test						
Real-time PCR *S. enterica*		99 (9.71%)	0	0	921 (90.29%)	1020 (100%)
Real-time PCR *S.* Enteritidis		20 (1.96%)	0	1 (0.10%)	999 (97.94%)	1020 (100%)
Real-time PCR *S.* Typhimurium		42 (4.12%)	0	1 (0.10%)	977 (95.78%)	1020 (100%)
Real-time PCR *S.* Virchow		24 (2.35%)	1 (0.10%)	1 (0.10%)	994 (97.45%)	1020 (100%)
PCR *S. enterica*		96 (9.41%)	2 (0.20%)	1 (0.10%)	921 (90.29%)	1020 (100%)
PCR *S.* Enteritidis		20 (1.96%)	0	1 (0.10%)	999 (97.94%)	1020 (100%)
PCR *S.* Typhimurium		42 (4.12%)	0	1 (0.10%)	977 (95.78%)	1020 (100%)
PCR *S.* Virchow		19 (1.86%)	4 (0.40%)	3 (0.29%)	994 (97.45%)	1020 (100%)
Cultivating *S. enterica*		99 (9.70%)	0	0	921 (90.30%)	1020 (100%)

**Table 5:** Comparison of various tests for the detection of *Salmonella enterica* and its types *S.* Enteritidis*, S.* Typhimurium, and *S.* Virchow.

	Result	SN, at 95% CI	SP, at 95% CI	PPV, at 95% CI	NPV, at 95% CI	Diagnostic Efficacy
Test						
Real-time PCR *S. enterica*		100	100	100	100	100
Real-time PCR *S.* Enteritidis		95.23 (93.93–96.53)	100	100	99.90 (99.71–100)	99.90
Real-time PCR *S.* Typhimurium		97.67 (96.77–98.57)	100	100	99.90 (99.71–100)	99.90
Real-time PCR *S.* Virchow		96.00 (94.8–97.2)	99.90 (99.71–100)	96.00 (94.8–97.2)	99.90 (99.71–100)	99.80
PCR *S. enterica*		98.97 (98.35–99.59)	99.78 (99.50–100)	97.95 (97.15–98.75)	99.89 (99.69–100)	99.71
PCR *S.* Enteritidis		95.24 (93.94–96.54)	100	100	99.90 (99.71–100)	99.90
PCR *S.* Typhimurium		97.67 (96.77–98.57)	100	100	99.89 (99.69–100)	99.90
PCR *S.* Virchow		86.36 (84.26–88.46)	99.60 (99.30–99.90)	82.61 (80.31–84.91)	99.70 (99.40–100)	99.31
Cultivating *S. enterica*		100	100	100	100	100

SN—Sensitivity; SP—Specificity; PPV—Positive Predictive Value; NPV—Negative Predictive Value; 95% CI—95% confidence interval.

**Table 6:** Typing of *Salmonella enterica* strains by RAPD PCR.

Serovar	Food Product				Clinical Sample			
	Group	Number of Isolates	Number of Amplicons	Size of Amplicons, bp	Group	Number of Isolates	Number of Amplicons	Size of Amplicons, bp
*S.* Enteritidis	A	8	6	250, 350, 650, 1000, 1250, 3000	A	13	6	250, 350, 650, 1000, 1250, 3000
*S.* Typhimurium	B	12	4	250, 350, 1000, 1250	B	29	4	250, 350, 1000, 1250
	C	2	3	250, 350, 1000				
*S.* Virchow	D	3	3	200, 650, 1200	I	19	3	300, 650, 1200
					F	2	2	400, 650
					G	2	4	300, 500, 650, 1200

## Data Availability

The data presented in this study are available on request from the corresponding author.
